# Designing prototype rapid test device at qualitative performance to detect residue of tetracycline in chicken carcass

**DOI:** 10.14202/vetworld.2022.1058-1065

**Published:** 2022-04-25

**Authors:** Mochamad Lazuardi, Eka Pramyrtha Hestianah, Tjuk Imam Restiadi

**Affiliations:** 1 Veterinary-Pharmacy Science Subdivision, Faculty of Veterinary Medicine, Universitas Airlangga, Mulyorejo rd. “C” Campus Universitas Airlangga, Surabaya, 60115, Indonesia; 2 Veterinary Histology Subdivision, Faculty of Veterinary Medicine, Universitas Airlangga, Mulyorejo rd. “C” campus Universitas Airlangga, Surabaya, 60115, Indonesia; 3 Veterinary Reproduction Division, Faculty of Veterinary Medicine, Universitas Airlangga, Mulyorejo rd. “C” Campus Universitas Airlangga, Surabaya, 60115, Indonesia

**Keywords:** antimicrobial resistance, color indicator, health lifestyle, light-emitting diode, precipitate principles

## Abstract

**Background and Aim:**

Human health problems due as a microbial resistance or tumors and cancers because consumption of the carcasses containing residues of tetracycline are main global problems in the context of fight against antimicrobial resistance phenomena. Explanation of the sustainable development goals, particularly point 3, is well recognized that all animal products for human consumption must be safe to live a healthy life. This study aimed to design a prototype of rapid test devices (RTD) based on principles of precipitate to obtain a specific color change after the process of reactions as an indicator to determine tetracycline residues in the carcass.

**Materials and Methods:**

Five samples of tetracycline-containing poultry carcasses using artificial add the tetracycline at pharmaceutics grade were examined using a prototype of a strong reaction solution for tetracycline fixation based on the concept bonded by ion Fe(III) at atom O in position atom C-1 at the ring of tetracycline and ion N^+^ as the functional branch of tetracycline. RTD detection was evaluated using a yellow color presentation and an absorbance spectrometric technique at a wavelength of 273 nm.

**Results:**

The following chemicals were used to create the best-fixed tetracycline residue: HCl and H_2_SO_4_ dissolved in H_2_O, chromatographic grade of 0.1 N and 0.5 N of HNO_3_, and 1% Fe (III) Cl. The RTD had a higher limit of detection (LOD) than the ultraviolet-visible spectrophotometer.

**Conclusion:**

The results of this study revealed that RTD, as constructed in this study, can be used to detect residue at LOD 44.764 mg/mL during 120 min of exposure through a light-emitting diode at 980 nm wavelength (p<0.05). The necessity for using RTD was because of the apparent limitations of conventional devices.

## Introduction

The livestock subsector is the fastest-growing in producing animal food, accounting for over 70% of the entire supply of human food products to meet animal protein demands [1,2]. In addition, human physical development necessitates a variety of proteins and minerals derived from fresh animal products. Therefore, it is challenging to supply animal-derived food with a healthy quality. The critical issue in producing a healthy product is ensuring it is devoid of chemical residues such as veterinary medications, herbicides, and insecticides [3-5]. In principle, all countries have stringent restrictions on the presence of hazardous substances in animal-sourced food and beverage items. Everyone who analyzes the influence of residues on health, which will have a less beneficial impact on health, understands this idea. Furthermore, all chemical residues in food and feed must be zero, according to the World Health Organization [6,7].

Point 12 of the sustainable development goals (SDGs) states that hazardous substances in the environment and food systems must be avoided at all costs. These dangerous compounds have several negative consequences for human health. Furthermore, the cost of rehabilitation to restore the condition of a body that has developed anomalies due to exposure to dangerous chemicals is prohibitively high. Harmful pollutants can be found in whole animal drug residues or metabolites. Antibiotics, beta-agonists, hormonal, and chemotherapeutic medications are among the toxic residues found in animals. Antibiotics are a common issue among these four groups. It has to do with the antimicrobial resistance (AMR) problem and the needs of SDG, point 12 program, which has as its central goal a human being’s healthy lifestyle [8,9].

Antibiotics with a broad spectrum of activity against the germ target of action as protein synthesis inhibitors are a typical type of antibiotic. Between 2016 and 2018, the usage of tetracycline for therapeutic action in the Asian Pacific region was estimated to be between 75% and 80%, with chicken therapy being the most common [10,11]. Tetracycline is also commonly used in aquatic breeding, with success rates ranging from 50% to 69% [12,13]. The widespread use of tetracycline in cattle will result in tetracycline residues that are difficult to detect. Milk and eggs are examples of food and beverage goods implicated. The formation of AMR, the interaction of tetracycline components with Fe (III) Chloride in the body, causing blackening of children’s teeth, or through the milk and dairy products are all health hazards associated with the presence of tetracycline residues.

The identified risks can be prevented if tetracycline residues in meat, milk, and eggs are tested [14,15]. The use of a special rapid test device (RTD) in examining tetracycline residues in fresh products of animal origin is still unavailable.

The principle of this research were designed to capture the specific reaction of tetracycline nuclei bound by element FeCl_3_ to produce yellow solutions [16,17]. The hypotheses of this research are hinged on the following statements; prototype of RTD is useful for detecting residue compared to spectrophotometer ultraviolet-visible (UV–Vis). This study aimed to design a prototype of RTD based on principles of precipitate to obtain a specific color change after the process of reactions as an indicator to determine tetracycline residues in the carcass.

## Materials and Methods

### Ethical approval

This study did not use any experimental animals. Hence, ethical approval did not require in this study. 

### Study period and location

This study was conducted from March 2020 to September 2020. The design of prototype of RTD and application of RTD was carried out at the Research Centre of Sub-division Veterinary Pharmacy Science, Faculty of Veterinary Medicine Universitas Airlangga.

### Chemical reagents and instrumentation

Tetracycline certified material reference (CRM) was purchased from Sigma-Aldrich (catalog no. 31741, lot #BCBW2124). In addition, other chemicals in level pro analysis grade were H_2_SO_4_, HNO_3_, HCl, and FeCl_2_. Aqua was used aqua pro chromatography Liquid chromatography-mass spectrometry grade from Merck KgaA, Germany, as present in catalog Z0476733 74G.

Spectrophotometer UV–Vis used the Genesys 10 from Thermo-scientific, Germany. pH meter Benchtop Mettler Toledo pH-016. Counterfeit money detector by UV at 365 nm and anti-stoke (980 nm wavelength) light-emitting diode (LED) lamp, with magnifying glass model Newmark EU-2038 production by Zhejiang Semtom Electronic Co., Ltd, China.

### Research design and research implementation

The study was conducted as an experimental post-test only control group design, with the following two-step research implementation: Step 1, optimization - validation by spectrometric technique, and Step 2, detecting the presence of tetracycline in animal carcass using RTD [18-20]. Detection of tetracycline in meat was calculated using Eq. 1 below [21,22].


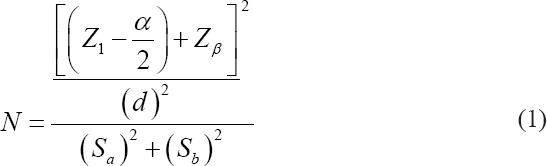


where {*Z*_1_ − (α/2)} at 1.96, with a significance of 0.05; *Z*_β_ at 1.645 by error limit of 5%; *d* at 3.62; *S_a_* at 1.7 and *S_b_* at 1.4 [23,24]. As the *N* value was rounded to 5, the control group comprised 5 data; the test group contained 5 data [25].

### Optimization and validation

The reagent of H_2_SO_4_ 0.1 N was prepared from 1 N stock H_2_SO_4_ with valence two and purity 95-97% at d 1.84 g/mL and MW 98.08 g/mol, then dilution with water to 10 times folds. HNO_3_ 0.5 N was prepared from the stock of HNO_3_ valence one and purity 98-99% at d 1.40 g/mL and MW 63.01 g/mol, then dilution with water to 28.8 times folds. HCl 0.1 N was prepared from 1 N HCl valence one and purity 37% at d: 1.19 and MW 36.5 g/mol then diluted with water to 10 folds. Furthermore, FeCl_3_ was prepared in fresh conditions at 1% (w/v), concentrated, dissolved in water then kept in a dark bottle. Serials tetracycline CRM (mg/mL) 0.5 mL of the 0.001; 0.002; 0.005; 0.010; 0.025; 0.052; 0.104; 1.04; 5.2; 10.4; 20.8; 26; 52; 73; 104; 118.45; 135.2; 145.6; 156; and 208 was dissolved in 0.5 mL of HCl. After that, the diluent was introduced into 0.5 mL of H_2_SO_4_ and HNO_3_, vigorously shaken for 5 min, and then added into FeCl3 0.5 mL as an active bounding reagent with yellow as the indicator color. The serial work on the tetracycline was executed 5 times. For the last step, all reagents were measured level of acidity by pH meter, followed by observation of absorptivity based on absorbance values at 273 nm (A_273_). Validation of analysis was carried out using a linearity test, precision test, limit of detection (LOD) test, and the limit of quantification (LOQ) test [26,27]. The accuracy analysis was evaluated by observation of recovery relative *(R)* values at serial tetracycline CRM (mg/mL) in three replicate carcasses as follows; 0.01; 0.1; 1; 5; 10; 15; 20; 25; 50; 75; 100; 150; and 200. All serial samples were prepared and then observed using the spectrometer to obtain percentage ratio recovery between absorbance samples to absorbance CRM dissolved in HCl. LOD was assessed by observed low concentrations of tetracycline but still detected at small absorptivity. The tetracycline was then diluted in HCl to a higher concentration than the lowest absorbance reading. The dilution in question was done in three concentration points, each of which was tiered as concentration increased. Next, a linear regression equation was made between X=concentration Y=absorbance. The regression equation was entered with the least absorbance value to calculate the detection limit. The detection limit value was multiplied by three to determine the quantification limit.

### Step two: Determination of tetracycline residue using RTD

The LOD for RTD was determined, and after that, the residues were detected by RTD. Five carcass samples were added through serial dilution of tetracycline with the following substeps. First, a solvent of tetracycline at 500 μL was introduced into the control group, which contained the carcass.

**Figure-1:**
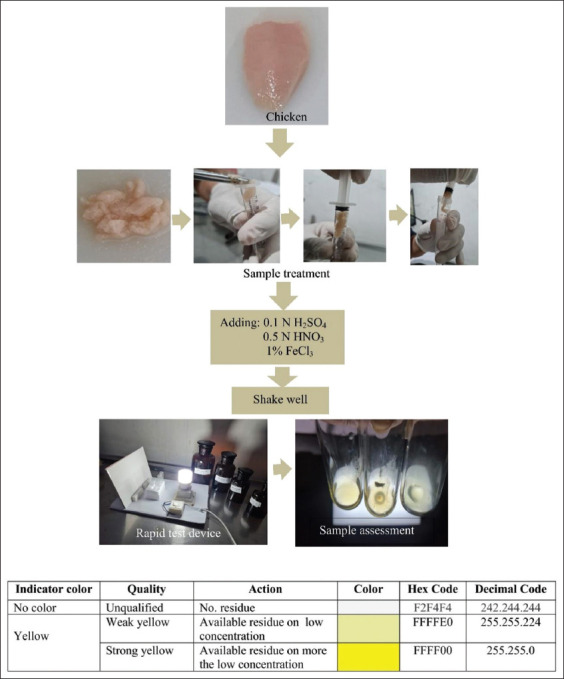
Protocol research and criteria indicator color of rapid test Devices on expose light-emitting diode and HyperText Markup Language code.

Substeps one: About 1 g of the carcass was weighed and pulverized to perform as a smooth meat product. Substep two: About 500 μL serials of tetracycline, starting from the lowest concentration referring to the LOD were added gradually as follows; 0.003; 0.1; 1; 10; 25. 30; 40; 45; 50; and 75 μg/mL. Substep three: The samples were vigorously shaken and allowed to stand for 24 h and afterward stored in the refrigerator. Substep four: The smooth meat product was inserted onto the barrel of a syringe having a 5 μL capacity without a needle. After that, the pressure on the top of the plunger until the smooth meat out from Luer inlet and collected in a centrifuge tube, then following centrifugation at a relative centrifugal force of 1500× *g* at 10 min. Substep five: The supernatant was allowed to cool on a glass tube of 5 mL. Substep six: After that, 0.1 N H_2_SO_4_ 500 mL, 0.5 N HNO_3_ 500 μL, and 1% FeCl_3_ 500 μL were added to the supernatant and the tube glass was closed by a rubber stopper, then followed by vigorous shaking for 3 min until the yellow color formed (approximately at wavelength 565-590 nm). Substep seven: The yellow color was observed using the detector on exposure LED lamps as the modified Indonesia Patent Registered P00202107382 on September 9, 2021. The limit detection of RTD was evaluated based on the yellow color formation of the samples at the lowest concentrations of serial tetracycline dilutions [28,29]. Criteria of the color yellow and hypertext markup language code are as follows as presented in Figure-1 [30-32]. The yellow color should be tested stable each 30 min, 60 min, 90 min, and 120 min to adequately observe the absence of the color, especially at high concentrations of tetracycline.

### Statistical analysis

In this analysis, 50% endpoint of the test group was determined using probit analysis at a 5% level of significance. In addition, a hypothesis test was carried out using an independent t-test between the test groups of RTD versus the test group of spectrophotometer UV–Vis at a 5% level of significance. The statistical analysis was executed using Statistical Package for the Social Sciences 24.0 software(IBM Corp., NY, USA).

## Results

Tetracycline was dissolved in 0.1 N HCl containing 0.1 N H_2_SO_4_, 0.5 N HNO_3_, and 1% FeCl_3_ and was obtained at a pH range of 1.80-2.30 with a 5-7 min stability (room temperature 22°C humidity 20%). A solution of FeCl_3_ must be made under new conditions since FeCl_3_ is a hygroscopic mineral that dissolves in water (v/v) (stability at a range of 20-24 h). The maximum wavelength of the visible domain was 273 nm in yellow color with pH 1.14-1.16 due to the research.

**Figure-2:**
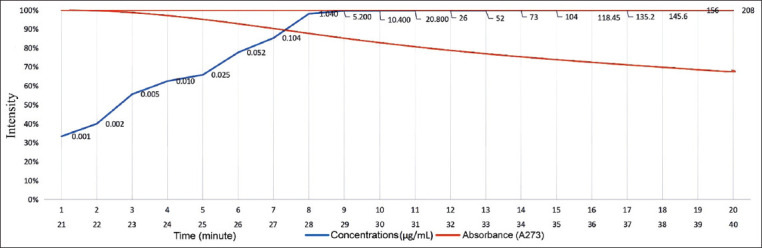
Stability test of intensity absorbance of tetracycline certified reference material on ranging 0.001-208 μg/mL in 0.1 N HCl (w/v) during the 40 min observed.

The linearity and precision at fifth replication of each concentration ranged between 0.001 μg/mL and 208 μg/mL (± standard deviation [SD] 0.001-0.002). Tetracycline CRM dissolved in 0.1 N HCl at range of absorbance (A_273_) 0.002–0.208 (±SD 0.001–0.002) demonstrated that response detector of each concentration showed strong correlations in R^2^ of 0.957 or equation Y=−4.793+962.094X and coefficient variation (CV) between 0.1% and 5.0%. More specifically, stability tests for absorbance values ranging from 0.001 g/mL to 208 g/mL were performed at intervals of 10-20 min (100% intensity), with a steady reduction in intensity from 21 to 40 min (Figure-2). By analyzing relative recovery (R %) of the three sample replications (n_1_ to n_3_), the accuracy of the research result in carcass matrix was discovered to be fairly excellent, as follows; 0.01 μg/mL in mean of absorbance 0.006±0.0005 (A_273_) at 85.714 (R %); 0.1 μg/mL in mean of absorbance 0.018±0.0005 (A_273_) at 94.737 (R %); 1 μg/mL in mean of absorbance 0.021±0.0006 (A_273_) at 95.454 (R %); 5 μg/mL in mean of absorbance 0.023±0.0006 (A_273_) at 95.833 (R %); 10 μg/mL in mean of absorbance 0.033±0.0007 (A_273_) at 97.059 (R %); 15 μg/mL in mean of absorbance 0.037±0.0005 (A_273_) at 97.368 (R %); 20 μg/mL in mean of absorbance 0.042±0.0006 (A_273_) at 97.674 (R %); 25 μg/mL in mean of absorbance 0.059±0.0005 (A_273_) at 98.33 (R %); 50 μg/mL in mean of absorbance 0.098±0.0005 (A_273_) at 100 (R %); 75 μg/mL in mean of absorbance 0.113±0.0006 (A_273_) at 99.123 (R %); 100 μg/mL in mean of absorbance 0.151±0.0005 (A_273_) at 99.561(R %); 150 μg/mL in mean of absorbance 0.169±0.0006 (A_273_) at 99.412 (R %); and 200 μg/mL in mean of absorbance 0.203±0.0006 (A_273_) at 99.024 (R %). The least concentrations could be observed in 0.011 µg/mL (0.006 A_273_), then by other concentrations of 0.022 µg/mL (0.023 A_273_) and increasing concentrations at 0.22 µg/mL (0.034 A_273_) led to the linear regression equation as follows; Y=0.013+0.099X at coefficient correlation (r)=0.828. The estimated LOD by inserting the least absorptivity at positive values of X (0.013 A_273_) was 0.00351 μg/mL, and then LOQ was calculated from 3 times of LOD, and resulted 0.0105 μg/mL. Following that, observation of limit detection for RTD analysis revealed the presence of the yellow color with a faint yellow color quality at tetracycline concentrations between 45 μg/mL and 50 μg/mL (Table-1). The 50% end point of concentrate for both of them was 44.764 g/mL, according to further analysis. The following color interpretations are provided below.

**Table 1:** Observation limit of detection and detected tetracycline pharmaceutic grade in samples using rapid test devices at five samples.

Concentrate (mg/mL) (*X*)	Test group by the rapid test device		Test group by spectrophotometer ultraviolet visible (A_273_)		p-value
	
	Carcass not detected/detected	Length of stability color (min)	Carcass not detected/detected	Length of stability absorbance (min)	
	
		30-120		30-120	
0.003	5/0	Not clear	0/5	Decreased	<0.05
0.1	5/0	Not clear	0/5	Decreased	
1	5/0	Not clear	0/5	Decreased	
10	5/0	Not clear	0/5	Decreased	
25	5/0	Not clear	0/5	Decreased	
30	5/0	Not clear	0/5	Decreased	
40	5/0	Not clear	0/5	Decreased	
45	3/2	Weak Yellow	0/5	Decreased	
50	0/5	Weak Yellow	0/5	Decreased	
75	0/5	Strong yellow	0/5	Decreased	

## Discussion

**Figure-3:**
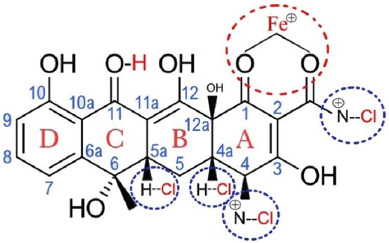
The ring structure of tetracycline dissolved in 0.1 N HCl and added with 1% Fe(III) Cl, and atom ferric bonded in the red ring, then Cl bonded in the blue ring.

The normalization design, percent, and volume of reagent for the chemical reaction to tetracycline as the principal ingredient of RTD were all found to be adequate. This process produced a residue attaching to the ring tetracycline and its functional groups. Tetracycline’s rings structure is divided into two parts: An upper easy to modify region and a lower difficult to modify region. The C-number 10 as a phenol, as well as a C-number 11 and C-number 12 as a keto-enol substructure in conjugation by 12a-OH groups and a number carbon between C-1 and C-3 as a diketo substructure, make up the functional branch of tetracycline as active bonds [33-35]. The outcome of this research extends the utility of the non-classical precipitate reaction from the positive charge of Fe (III) Cl to tetracycline (Figure-3).

**Figure-4:**
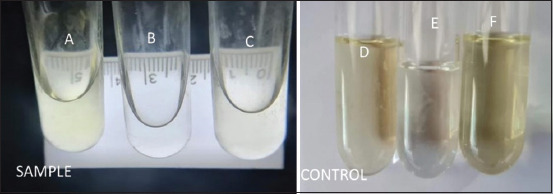
The quality yellow color solution of the rapid test device from the carcass (sample): (a) Intense yellow, (b) not clear, (c) weak yellow. Control: (d) weak yellow, (e) blank color, (f) intense yellow color.

The UV absorption spectra were observed as a conjugation bond at region characteristic A_273_ of the ABCD-ring system in this analyte. The Fe (III) atoms were linked in the red ring. On C-4, C-4a, and C-5, the chloride atom was connected at the blue ring (Figures-3 and 4). The function groups coupled with the chloride atom in a complex tetracycline bound to Fe (III) will give off a yellow color [36]. The investigation showed that 0.1 N H_2_SO_4_ and 0.5 N HNO_3_ prevented the impurities reaction to the ring of red and rings of blue. Hence, the addition of FeCl_3_ produced a yellow color indicator. At greater than 47.746 μg/mL of leftover tetracycline, the appearance of the yellow hue can be seen using human eyes, and it may be further clarified by LED light. Compared with the observation made by spectrophotometer UV–Vis, the design of strength of normality and percent of reagent were appropriate for the test with linearity at R^2^ closed to 1.00 with CV precision ≤5% [34,37,38]. The highest percentage of CV was found in the smallest concentration, which can be explained that smaller concentrations produced bigger variability. The absorptivity stability in a range of concentrations from 0.001 μg/mL to 208 μg/mL was roughly 20 min after adding the final solution, FeCl_3_ 1% (Figure-2). This is due to the high acidity of all reagents and the maximum concentration of oxidizing agent (O_2_+4H^+^) from air and H_2_SO_4_ and HNO_3_ reagents. The process of reducing analyte absorptivity was shown first because the top of the cuvettes could not be stopped at a precise level; therefore, O_2_ from the air was injected into the cuvettes [39]. It is well known that O_2_ may be obtained from H_2_SO_4_ and HNO_3_, but adding O_2_ from the air raises the concentration of the oxidizing agent. According to the accuracy evaluation based on spectrophotometer assessment, trueness values of relative recovery follow the Analytical Methods Committee (AMC) regulations, which are between 80 and 120% [40,41]. The knowledge of the validation technique has not been regulated for RTD; however, the LOD result can be translated to color intensity utilizing a portion of the validation method from AMC. The results of LOD’s investigation revealed that yellow at low levels was available in about 120 min. Colors observed for more than 120 min were not recommended. The unsteady color after 120 min is because the RTD reagent is at an acidic pH, which causes the yellow indicator color to fade even though the glass tube is still closed [42]. The sharpness of the yellow color is mostly determined by seven factors: (1) Adequate tetracycline concentration after separation from matrix biology, (2) The FeCl_3_ solution’s purity, (3) prepare the supernatant for performance by cleaning contaminants, (4) clean and clear all reagents, (5) clean and clear all hard surfaces at process preparations, and (6) clean and clear the tube glass for collected samples, and (7) exposure adequacy of LED lamps [43,44]. The production of pure tetracycline involves four steps, namely; (1) the animal is pulverized and then transferred onto a barrel of a syringe having a capacity of 5 mL without a needle, and then a slight pressure is applied at the top of the plunger until the smooth meat exits through the Luer inlet, (2) the compounds dissolved in 0.1 N H_2_SO_4_, are separated (3) The other compound dissolved in 0.5 N HNO_3_ are separated 4. The samples were centrifuged at 1500× *g* at 10 min. At the 4^th^ processing stage, the centrifuged sample was separated and extracted to obtain an adequate concentrate to bind Fe (III) Cl, resulting in the yellow color [45,46]. The purity of all reagents was important because contaminants by other compounds cause the bond between tetracycline and Fe (III) Cl loss. The water for dilution of all reagents must be at chromatographic grade; impurities in aqua will reduce the yellow color of indicator reagents (Figure-1). The LED lamp detector using visual detection with the eyes must be efficient when exposed samples. Hypothesis test by analysis independent t-test as presented in Table-1 showed that sample from the carcass could be detected compared to UV–Vis spectrophotometer, but concentration should be initiated at more than LOD of the RTD as designed (p<0.05). Compared RTD with references to establish the superiority of the work, as described in Table-2 [47-50].

**Table 2:** Comparison of the findings of the precipitation-based tetracycline rapid test devices with other rapid test devices for detection of tetracycline.

No.	Model of rapid test devices	Specificity test	Limit of detection (ppm)	Simplicity using indicators reading time after preparation (min)	Note and reference
1	Precipitation-based	Tetracycline	47.746	3	New prototype
2	Competitive lateral flow immunochromatographic assay	Tetracycline	Not clear	5-10	[47]
3	Immunoassay	Antibiotics	Not clear	5-15	[48,49]
4	Four-plate test	Flomocaine, Tetracycline, Sulfonamide, Enrofloxacin	Not clear	Not clear	[50]

The following are the limitations of RTD as a design in this study: (1) Cannot detect analyte with metabolite structure or incomplete molecule structure, (2) analyte on deep tissue of carcass, (3) residue at carcass more than 24 h slaughtered, (4) residue with the identical structure of tetracycline, (5) residue has been the reaction by other compounds to bonded the FeCl_3_ site, and (6) carcass protein is denatured. The limitation of these gadgets is that they must be utilized with caution.

## Conclusion

According to the findings, the RTD design for tetracycline detection in carcass was suitable for detecting residue at a concentration of more than 44.764 μg/mL on displayed by indicating yellow color during the 120 min period. Before adding the Fe (III) Cl, the RTD was useful when there was no contamination in the analyte.

## Authors’ Contributions

ML: Prepared and revised the manuscript, designed the experimental protocol, carried out application of RTD, and monitored application of RTD. EPH: Carried out sample preparations and edited the manuscript. TIR: Carcass preparations and monitored for positive samples. All authors have read and approved the final manuscript.
